# How should trial teams make decisions about the proportions and diversity of the ethnic groups in their trial?

**DOI:** 10.1186/s13063-024-08625-5

**Published:** 2024-11-15

**Authors:** Shaun Treweek, Katie Gillies, Miles D. Witham, Declan Devane, Kamlesh Khunti, Peter Bower, Adwoa Parker, Irene Soulsby, Bārbala Ostrovska, Sarah Prowse, Heidi Green

**Affiliations:** 1 Aberdeen Centre for Evaluation, University of Aberdeen, Aberdeen, AB25 2ZD UK; 2 AGE Research Group, Faculty of Medical Sciences, NIHR Newcastle Biomedical Research Centre, Newcastle Upon Tyne Hospitals NHS Foundation Trust, Cumbria Northumberland Tyne and Wear NHS Foundation Trust and, Newcastle University, Newcastle Upon Tyne, UK; 3 HRB-Trials Methodology Research Network, School of Nursing and Midwifery, University of Galway, Galway, Ireland; 4 Diabetes Research Centre, Centre for Ethnic Health Research, NIHR ARC East Midlands, University of Leicester, Leicester, UK; 5 NIHR ARC Greater Manchester, University of Manchester, Manchester, UK; 6 York Trials Unit, Department of Health Sciences, University of York, York, UK; 7 COUCH Health, Manchester, UK

**Keywords:** Ethnicity, Equity, Diversity and inclusion, Recruitment, Retention

## Abstract

**Background:**

The benefits of randomised trials are not shared equally, and people from ethnic minority groups are a key constituency under-served by clinical research and clinical care. The STRIDE project aimed to give trialists practical information about how to decide which ethnic groups should be in their trials, and at what proportion.

**Methods:**

We considered trials in six clinical areas: cancer, cardiovascular, diabetes, maternal health, mental health, and smoking cessation. We created a summary for each, including participants–intervention–comparators–outcomes, and data on disease prevalence by ethnicity. These were discussed with panels with clinical expertise, trial and methodology expertise, lived experience, funding, and experience of working with and on behalf of ethnic communities. For each trial, we asked panel members to decide which ethnic groups should have been involved and at what proportion.

**Results:**

We discussed 23 trials with 40 individual panel members. Panels found our questions difficult to answer. The lack of publicly available data on prevalence by ethnicity was central to this. Where data were available, decision-making was easier but not simple.

The discussions led to eight STRIDE recommendations. We recommend that discussions involve diverse teams and that discussions need time, with access to the best available data. In the absence of data or consensus, we recommend the adoption of ‘default’ minimum rates of inclusion, with oversampling considered. These discussions should inform site selection, and the practical challenges of recruitment and retention should not determine which groups are to be included.

We also suggest five policy initiatives to support implementation of the recommendations. Broadly, these are (1) funders need to signal that ethnic diversity is expected, (2) trial teams need access to better data, (3) funders and others need to signal that ethnic diversity means better science, (4) more funding is needed for evaluation, and (5) Good Clinical Practice training should cover ethnic diversity.

**Conclusions:**

Agreeing targets for which ethnic groups to involve in a trial is essential but difficult. Our eight recommendations could help to make trials more ethnically diverse if followed, and we suggest five policy initiatives that would create a supportive environment for their implementation.

**Supplementary Information:**

The online version contains supplementary material available at 10.1186/s13063-024-08625-5.

## Introduction

Well-designed randomised trials are an important way to improve health and social care outcomes and population health. Their results can lead to the development of evidence-based guideline recommendations and the introduction of better treatments and models of care and help to stop the use of harmful or ineffective interventions.

These benefits are not shared equally. In 2020, the UK’s National Institute for Health and Care Research (NIHR) ‘Innovations in Clinical Trial Design and Delivery for under-served groups (INCLUDE)’ initiative acknowledged that many people do not benefit from health research because they are ignored or forgotten in the design, running, analysis, and reporting of research [1]. The authors presented the INCLUDE roadmap, an approach to trial delivery that aims to ensure improvement in the ‘quality, credibility, and applicability of research data and hence better healthcare delivery for a wide range of currently under-served groups’ [1].

People from ethnic minority groups are a key constituency under-served by clinical research and clinical care. It is not difficult to demonstrate this [2–4], but as an exemplar, consider maternal mortality. Maternal mortality in the UK is almost three times higher for Black women than it is for white women [5], but the current (2023) UK national guidance for intrapartum care (National Institute for Health and Care Excellence (NICE) Guidance NG235) only mentions ethnicity to say that no ethnicity-specific data are available [6]. Postnatal care and caesarean birth guidance are much the same [7, 8]. Caution is needed when including ethnicity in guideline recommendations to avoid unfounded assumptions about biological and cultural difference [9]. For example, guidance from NHS England on spirometry [10] recommends correction factors for a range of ethnic groups, despite the risk that this inappropriately labels asthma as controlled in Black children [11]. The 2014 NICE guidance on chronic kidney disease recommended adjusting glomerular filtration rate (eGFR), the most common test of kidney function, when used with Black people. This was removed from the 2021 guidance because of the risk of over-estimation of eGFR and subsequent inequality of care [12].

However, the almost complete absence of ethnicity-related considerations in maternity guidelines seems disconnected from the outcome inequality seen across ethnic groups [4, 13]. These omissions lead to recommendations and health and social care practice built on evidence that may not include the needs and perspectives of the ethnic groups that are often affected most. This is not primarily an issue of biological response (though it can be [14, 15]) but of access, implementation, and acceptability. This widens inequity.

That trials need to become more ethnically diverse is not disputed. Rather the key questions are:

1. How should we decide which ethnic groups to include in our trial?
2. What proportion of a trial population should be from these ethnic groups?

It is these questions that the STRIDE project aimed to answer.

This work is part of the Trial Forge initiative to improve trial efficiency (https://www.trialforge.org/).

## Methods

Our central approach was to create panels of people to look at real trials and discuss which ethnic groups those trials should have involved and at what proportion. There were two stages to this.

### Stage 1: Identifying the trials to discuss

We focused on recent trials in seven major clinical areas:

1. Bowel, breast, and prostate cancer
2. Cardiovascular disease
3. Diabetes (types 1 and 2)
4. HIV*
5. Lupus (systemic lupus erythematosus)*
6. Mental health
7. Smoking cessation

*As our search did not identify suitable HIV and lupus trials, we replaced them with maternal and infant health trials: see the ‘Results’ section.

We prioritised depth over breadth, i.e. we chose to focus on fewer clinical areas so that we could discuss more trials in those areas. The seven clinical areas were chosen because we expected variation in prevalence or severity in outcomes across ethnic groups in these areas. We focused further: most trials would be in the four areas of cancer, cardiovascular disease, diabetes, and mental health. Smoking cessation, HIV, and lupus were what we called ‘edge cases’; trial areas specifically chosen to give some breadth to our sample rather than depth. Trials were identified by searching the NIHR Journals Library (https://www.journalslibrary.nihr.ac.uk/#/) from 2015 onwards, supplemented by a search of the World Health Organization (WHO) Trials Registry Platform (https://www.who.int/clinical-trials-registry-platform) for non-NIHR trials, also from 2015. We completed an NIHR INCLUDE Ethnicity Framework for each included trial. This framework is a tool to help trial teams think about which ethnic groups should be included in a trial and what the challenges of achieving this might be [16].

### Stage 2: Creating and running the panels to discuss the trials

We wanted to create panels of people with clinical expertise, trial expertise, methodological expertise, lived experience of the clinical condition, experience of representing diverse ethnic communities in discussions about health, experience of funding trials, and health policy experience.

Potential panel members were suggested by the project team based on a combination of personal links, who was publishing research in the clinical areas, seminars, and talks we had heard, and recommendations from colleagues. Potential members were invited sequentially until we felt the panel adequately represented the perspectives of relevant parties (i.e. the panel had some patient, clinical, trial and methodological expertise).

All panel meetings were held online using Zoom and scheduled for 2 h. The cancer, cardiovascular disease, diabetes, and mental health trials were each spread over two panel meetings, while maternal health and smoking cessation had one meeting each. ST chaired all meetings, and all but two meetings were also attended by HG. Other STRIDE team members attended some meetings depending on their clinical, lived, or methodological expertise. Prior to the meeting, participants received a summary of the trials to be discussed and information on disease prevalence, severity, and progression (if relevant) by ethnicity, which we also talked through in the meeting. The information we provided to panel members prior to each of the ten discussions is available through Open Science Framework (OSF) at https://osf.io/jmqsx/.

### Analysis

Meetings were audio-recorded, and ST and HG also took notes. These notes were amended or supplemented after listening again to the audio-recording and ST and HG reached agreement on a final set of notes that provided an accurate and concise summary of the panel discussion for each trial. The notes contained information that was focused on the main task—which ethnic groups and at what proportion—as well as more general comments on increasing trial diversity. These were edited further to include just the comments focused on which ethnic groups the panel thought should have been involved and at what proportion, which were attached to the relevant INCLUDE Ethnicity Framework to go into the example set at https://www.trialforge.org/trial-diversity/include/.

The general comments, and the STRIDE team’s reflections on the panel discussions, were coded by ST using a directed content analysis approach [17], discussed with HG and then approved by the STRIDE team. These were then condensed into a set of recommendations about how trials teams can improve the ethnic diversity of their trials. These recommendations were agreed by the wider STRIDE team.

## Results

A total of 106 trials met our criteria, HG and ST narrowed this set of trials down to 21 (18 identified via NIHR, three via WHO) by considering trial interventions (e.g. drug, rehabilitation, surgery), design features (e.g. primary care, secondary care, individually randomised, cluster randomised), and the number of trials we planned to include in each clinical area. We selected a group of trials that had a diverse mix of interventions and design features but was not so large as to make the online panel phase unfeasible.

The shortlist of selected trials was discussed and approved by the wider STRIDE team. Following consensus within the wider team, we included four cancer trials, six cardiovascular disease trials, four diabetes trials, six mental health trials, and one smoking cessation trial.

The search did not identify suitable HIV or lupus trials. We chose to replace these clinical areas with two maternal and infant health trials because of the well-known differences in maternal mortality across different ethnic groups [5]. We chose our two maternal health trials in a different way to the other trials. One was identified after discussion with colleagues working in maternal health and one through the Trial Forge team’s awareness of a trial with a design that had important ethnicity related considerations. The list of 23 trials is given in Supplementary file 1.

We held ten online panel meetings between 23 September 2022 and 28 November 2022. A total of 40 individual panel members from across the UK and Ireland were involved (see Table 1). Most members were involved in only one panel, but for conditions like cancer and diabetes where the trials were spread across two meetings, there was some overlap in membership across meetings. All but one meeting used the full 2 h allocated to complete discussions. One meeting had to finish around 30 min early because of participants’ other commitments, but this was known from the start and discussion was managed to use the time available effectively.

**Table 1:** The breakdown of participant roles at each of the ten meetings

	**Public contributor**	**Charity**	**Specialists in patient engagement/involvement**	**Health professionals**	**Public health professional**	**Trialist**	**Academic**	**Health network/advisory**
**Panel**								
Cancer 1	1		2^a^			1	1^a^	
Cancer 2	2^a^	1	2^a^			1	2^a^	
Cardiovascular 1	1						2^a^	
Cardiovascular 2	1	1	1^a^		1		3^a^	1
Diabetes 1	2	2	1^a^	2			2^a^	
Diabetes 2		1	1^a^	1			2^a^	
Maternal and infant	1		1^a^			1	5^b^	
Mental health 1		1	1				5^b^	
Mental health 2	1		2^a^				2^b^	
Smoking cessation	2		1^a^		1		2^a^	
**Totals**	**11**	**6**	**12**	**3**	**2**	**3**	**26**	**1**
**Notes**								

Some roles overlapped. For example, an academic could also have a clinical role, or a trialist could also be an academic. The main role for the panel is counted here. Thirty individuals attended just one meeting, eight people attended two meetings, one person attended eight meetings, and one person attended all ten meetings

^a^One was a STRIDE team member

^b^Three were STRIDE team members

### Deviation from our intended plan

For the first two panels (on smoking cessation and maternal and infant health), we provided a completed INCLUDE Ethnicity Framework for each trial. This led to time being spent discussing how different ethnic groups might be recruited, which was not the focus of our work. For subsequent meetings, we did not provide the completed Framework but instead gave background information in the disease and a brief Participants–Intervention–Comparator–Outcomes (i.e. PICO) description for each trial, which we talked through in the meeting.

### The eight STRIDE recommendations

Panel comments broadly fell into one of three categories:

1. Comments on the trial being discussed
2. General comments on the ethnic groups to be involved in trials in their respective disease area
3. Comments on the process of how to consider which ethnic groups to include in a trial and at what proportion

**Fig. 1:**
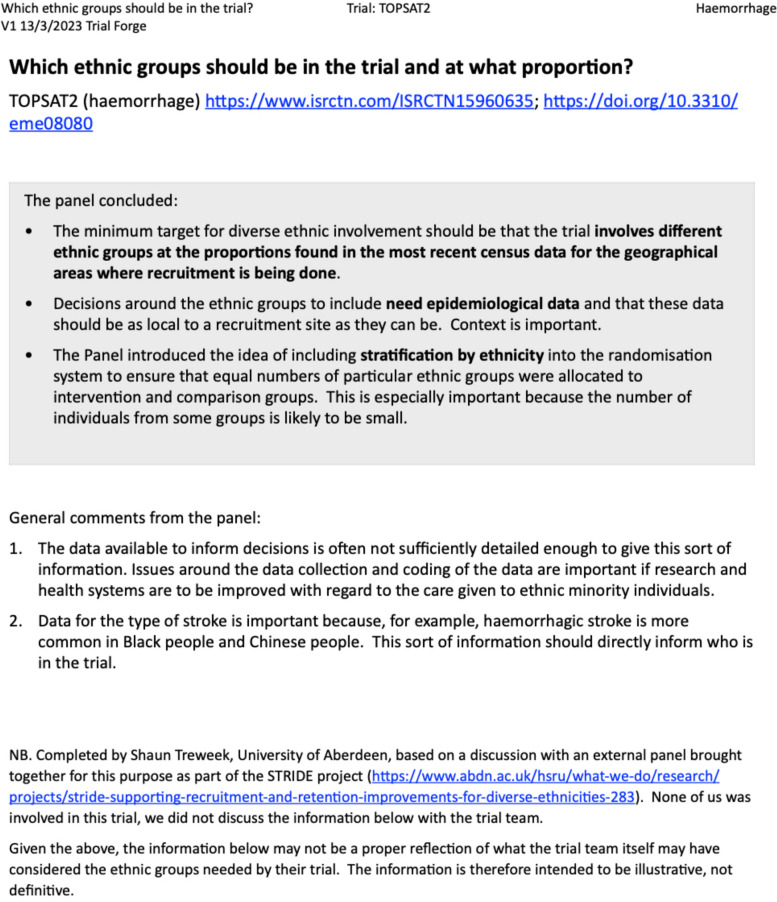
An example of an edited summary of a panel discussion about a trial, this one for TOPSAT2 (https://doi.org/10.1186/ISRCTN15960635), a trial of two approaches to treating strokes caused by spontaneous subarachnoid haemorrhage

Our initial (now anonymised) notes from each trial discussion are available through OSF https://osf.io/jmqsx/. With one exception, these notes were edited by ST to give a concise summary of a panel’s thoughts for the INCLUDE Ethnicity Framework example set at https://www.trialforge.org/trial-diversity/include/ and are also at https://osf.io/jmqsx/. The exception is the UNI trial, which was part of the maternal and infant health panel discussion. UNI was not funded, and because all the other trials in the example set were completed, we decided against putting UNI into the example set. Figure 1 is an example of an edited summary, this one for TOPSAT2 (https://doi.org/10.1186/ISRCTN15960635), a trial of two approaches to treating strokes caused by spontaneous subarachnoid haemorrhage.

Panels found our questions difficult to answer. The main reason for this was the lack of trusted, publicly available data on disease prevalence, severity, and progression by ethnicity across various contexts (e.g. the UK). Where data were available, decision-making was easier but not simple. Crucially, there was often a lack of confidence that diagnostic data reflected true prevalence. For example, discussion of a prostate cancer trial led to concern that accepting existing data at face value on, say, treatment acceptability, risks hard-wiring disadvantage into research because of the lack of diversity amongst those asked about acceptability. Intersectionality, and whether the participant characteristic to be targeted by a trial should be ethnicity, or ethnicity in combination with other characteristics such as socioeconomic disadvantage or age, was raised many times, though without clear solutions.

Panel comments were collected into a single document and coded by disease area and panel. The STRIDE team also added its reflections on the process of considering which ethnic groups to be included in a trial. This document is in Supplementary file 2.

The coding document and our reflections on the panel discussions led us to eight STRIDE recommendations (Table 2). A longer version of the recommendations document is available in Supplementary file 3 and at https://www.trialforge.org/trial-diversity/how-to-decide-which-ethnic-groups-your-trial-needs/.

**Table 2:** The eight STRIDE Recommendations

**The eight STRIDE recommendations**	
The recommendations in full are at https://www.trialforge.org/trial-diversity/how-to-decide-which-ethnic-groups-your-trial-needs/. See Supplementary file 2 for source information recording coding	
**Trials teams need to be more diverse**	**Trial teams need the perspectives of people from diverse ethnic communities with lived experience of the condition or disease**
Trial teams should themselves aim to be more diverse, not only in terms of ethnicity but other characteristics too. Seeing researchers from your own community reassures many members of the public that research is relevant to them	Having ethnic diversity amongst patient and public contributors with lived experience is essential. These individuals speak with authority on both the condition but also how people from their ethnic group view the condition, treatment options and the proposed trial. These perspectives are crucial if the trial is to be inclusive
*(Main source: Cardiovascular panel 2, comments 12 and 13)*	*(Main source: STRIDE team reflections, comment 1)*
**Discussions need time**	**Discussions are more productive with data on disease or condition prevalence, severity and progression by ethnicity**
We would suggest a minimum of two hours, which allows time to introduce the trial (we found using PICO (Participants–Intervention–Comparator–Outcomes) worked well for this), the disease or condition and its implications and the data around disease prevalence, severity and progression by ethnicity	Discussions about which ethnic groups to involve in a trial are more straightforward when informed by data on disease or condition prevalence, severity and progression by ethnicity
*(Main source: STRIDE team reflections, comment 4)*	*(Main source: STRIDE team reflections, comment 3)*
**Don’t think about the practicalities of how to recruit and retain people until later**	**If in doubt, use the STRIDE defaults for ethnic group inclusion**
Thinking about the practicalities of how to recruit and retain people is a question for later: the question for now is which ethnic groups need to be in the trial. Researchers in trial teams tend to think about the practicalities of recruitment and retention immediately but that isn’t what this stage of trial design is about	Where a team cannot reach a conclusion with regard to the ethnic groups needed, or perhaps lacks the time and/or resources to have a full discussion, the STRIDE panel discussions suggest adopting the following default inclusion position: a. The minimum target is that ethnic groups are included at the same proportion as is found amongst the population of people with the condition targeted by the trial Where disease data by ethnicity do not exist, or cannot be obtained, the STRIDE panel discussions suggest adopting the following default inclusion position: a) The minimum target is that ethnic groups are included at the same proportion as is found in the most recent census data. A trial intending national reach should use national census data. A trial with more local reach could aim for census proportions in the relevant geographical areas
*(Main source: STRIDE team reflections, comment 2; Smoking cessation, comment 4)*	*(Main source: STRIDE team reflections, comment 5)*
**Consider over-sampling some ethnic groups**	**Think about ethnicity when choosing trial sites**
Involving some ethnic minority groups at proportions seen in the general population or at proportions seen for a particular disease or condition may mean that the number of individuals involved is still low The trial team could consider over-sampling these ethnic groups so that they are over-represented within the trial relative to their representation within the disease or condition, or the general population. This will increase the number of individuals from these ethnic groups in the trial and may improve the team’s ability to draw conclusions from their data	The ethnic groups a trial needs should influence decisions about where to place trial sites
*(Main source: Cancer panel 1, comments 10 and 12)*	*(Main source: STRIDE team reflections, comment 5)*

In summary, we recommend that discussions involve diverse teams including representatives from different communities with appropriate lived experience. Discussions should be given sufficient time, with access to the best available data. In the absence of accurate data or consensus, we recommend the adoption of ‘default’ positions specifying minimum rates of inclusion of different groups, with oversampling considered in certain contexts. Discussions should inform decisions about site selection, and we recommend that the practical challenges of recruitment and retention should not determine which groups should be included.

## Discussion

The need for trialists to include diverse ethnic groups in their trials is widely recognised [1, 14, 16, 18–20] and sometimes mandated [21, 22]. The start of designing an ethnically diverse trial is being clear about which ethnic groups need to be in the trial to answer its research question and at what proportion. As our panels found, these are not easy questions to answer.

Indeed, a limitation of our work is that we were generally unable to say in concrete terms which ethnic groups should have been involved in the trials we discussed or at what proportion. With two exceptions (the two maternal and infant health trials), our panels were not discussing trials they knew from personal experience. Instead, panel members depended on the summaries we provided and their own knowledge and experience. It is possible that working directly with trial teams may have led to different results. That said, in both cases where we had trial team members in our panel, the final target suggested was the census-based default that became part of our Recommendation 6. The full reports we used for the trials, mostly substantial NIHR reports, did not generally present detailed discussion of how ethnicity affected trial design, although PROPELS, one of the diabetes trials, was an exception [23]. This suggests that having more trial team members on our panels would have been unlikely to change our conclusions or recommendations.

### Policy initiatives to support implementation

The eight recommendations shown in Table 2 will, if implemented, help to make trials more ethnically diverse. The rub is the qualifier—*if implemented*. Making recommendations is easy; implementation is where things get difficult. Trial teams, both in academia and industry, can start using the recommendations in Table 2 immediately. Some, such as not worrying about the practicalities of how the team will recruit and retain people until after you have identified the groups you need (Recommendation 5) or thinking about ethnicity when choosing trial sites (Recommendation 8), are relatively easy to implement. Others, such as the need for more diverse trial teams (Recommendation 1), are system-level challenges, although research teams need to have this in mind when training and hiring staff.

We, the wider Trial Forge team, and others are disseminating these ideas to trialists and others, most recently at the 2024 International Clinical Trials Methodology Conference (https://ictmc.org), including through KK’s keynote address. However, this type of dissemination is unlikely to be enough. Below, we outline five policy initiatives that we think would provide an environment in which our recommendations would have a greater chance of improving the ethnic diversity of trials. Without these initiatives, change will be slow and the ability of trial teams to make their trials ethnically diverse will be hampered. Some of the policy initiatives are already underway in whole or part in some jurisdictions, giving reason for optimism. However, implementation of these initiatives needs to be accelerated. It should also be noted that while we focus on ethnic diversity, we are confident these policy initiatives will support greater diversity in trials more generally.

#### Policy 1: Financial signalling by funders

To generalise, many ethnic groups have a low level of trust in trials and research, and trial teams have a low level of knowledge in how to engage effectively with people in these groups. Given this, everyone involved in the process of funding and delivering trials needs to accept that ethnically diverse trials will take longer and cost more than trials that are less inclusive. This may change as trust increases and evidence about how to engage effectively improves.

Funders need to clearly signal to trial teams that the time and costs needed for greater ethnic diversity *are not only accepted but also expected*. The expectation part is important: funders need to signal that greater ethnic diversity is not optional, but a requirement. Public acceptance by the funder of the greater time and cost this entails is a necessary consequence of the requirement.

An exemplar of the importance of signalling is support by NIHR, a UK funder, for Studies Within A Trial (SWATs) [24]. These evaluations of trial process alternatives were largely limited to a few enthusiasts in the UK until NIHR signalled the wider need for SWATs by offering specific funding of £10,000 and, more recently, £30,000 per SWAT. Other funders such as the Irish Health Research Board and the Canadian Accelerating Clinical Trials program signal their encouragement through similar schemes, the latter funder offering nearer to £57,000 for a SWAT. *Explicit* signalling is needed to give trial teams the confidence to take on the time and costs of making trials more ethnically diverse.

#### Policy 2: Health and social care providers need to routinely collect diversity-related data, and these data need to be made available to trial teams

The first question any trial team designing an ethnically inclusive trial asks is how is disease prevalence, severity, and progression spread across different ethnic groups.

Finding data to answer this question is hard. For example, our panel discussing PROSPER (https://www.isrctn.com/ISRCTN35358984), a UK trial of exercise to prevent shoulder problems in patients undergoing breast cancer treatment, wanted data on the ethnicity of people who get shoulder problems during breast cancer treatment. The PROSPER trial team did not mention ethnicity when discussing shoulder pain [25] and neither did a systematic review of shoulder and arm pain in patients after breast cancer treatment [26]. In a country as diverse as the UK, with around 20% of the population being part of an ethnic minority group, it seems unlikely that the 8% ethnic minority involvement in PROSPER reflects the clinical population of people with shoulder problems during breast cancer treatment. PROSPER is not a special case: our panels struggled with limited or lack of data for all the trials we discussed.

The UK Health Data Research Alliance Ethnicity Coding Standard Special Interest Group, which KK co-chairs has already called for ethnicity data and other determinants of health to be routinely and consistently collected across the health and social care sectors [27]. We agree—collecting these data needs to become standard policy. Anonymised versions of these data then need to be made easily available to those planning and commissioning trials; otherwise, trial teams will be designing in the dark.

Which data to collect is a non-trivial question, and groups including the UK Health Data Research Alliance are working to answer it. A reasonable starting point is the PRO EDI list of participant characteristics [28], a core information set developed by ST, DD, and others for anyone wanting to describe the characteristics of a population to support equity, diversity, and inclusion judgements.

#### Policy 3: Funders and other policymakers need to signal that ethnic diversity in trials leads to more useful science

Ethnic inclusion targets are essential; we suggest using ranges (e.g. Black British people: 5% to 8%) as a good way to present them. But targets generate a thorny question: what should we do if targets for some groups are being missed, while those for others are being exceeded?

This is a question about who brings most scientific value to the trial. Trial teams and funders need to accept that the answer in many cases will be to halt recruitment of those groups where targets are being exceeded to allow time for other groups to take their place in the trial.

There is precedent. ILANA, a UK human immunodeficiency virus study, had targets of 50% women, 50% ethnic minority, and 30% over the age of 50 and used recruitment caps to enforce these targets [29]. Moderna, a pharmaceutical company, deliberately slowed recruitment of white people during COVE, a US mRNA-1273 COVID-19 vaccine trial, to allow recruitment of under-served groups, especially Black and Hispanic/Latinx people [30]. It did so because ‘Moderna and the US [United States] government understood that achieving a diverse study population was essential to ensure a more robust and representative body of clinical knowledge’ [30].

The policy of financial signalling mentioned above should acknowledge that decisions to prioritise the involvement of some people over others is appropriate where greater ethnic diversity improves the scientific and decision-making value of the trial. Like many things in trials, this is a judgement, but current research policy from major funders and regulators explicitly highlights the importance of greater involvement of ethnic minority individuals in trials [21, 22, 31].

Moreover, as part of the approvals process, approval bodies such as ethics committees should review a plan submitted by the trial team that outlines the characteristics of people who will be in the trial and why, what the recruitment and retention targets are *by participant group*, how targets will be monitored, and what will be done if progress against targets is not as expected. This policy is already coming in the UK [21] (and to which ST contributed) and the US Food & Drug Administration, a regulator, started doing this in 2022 [22].

A table of who should be in the trial compared to who is being recruited and retained should be a mandatory part of progress reports to funders, trial steering committees, and data monitoring and ethics committees. In all cases, action from the trial team would be expected where important differences appear between who should be in the trial and who is in the trial.

Journals publishing trial protocols should mandate that the protocol include a table describing the expected characteristics of people that will be recruited for the trial and why. Journals publishing trial reports should ask for that table to be updated with who was recruited and retained. There should be an expectation that results will be interpreted by considering any differences. The New England Journal of Medicine started asking for something like this in 2021 [32].

Finally, to support interpretation across trials and to avoid research waste, the reporting policies above should aim for a high degree of consistency regarding the core set of characteristics that trial teams need to collect and report. The PRO EDI table is, we think, a reasonable starting point [28].

#### Policy 4: Funders need to put more money into evaluation

The ability of trial teams to use evidence-informed recruitment and retention strategies to support ethnically diverse trials is hamstrung by there being almost no robust evidence on which to base those strategies [33, 34]. The current evidence on how to do ethnically inclusive trials is woefully inadequate and this urgently needs to change.

Some of us have previously suggested that trial funders should put 10% of their funding into applied methodology research and supporting infrastructure [35]. Lack of funding for methodology research, dissemination, and infrastructure is leading to research waste [35–38], of which insufficiently diverse trials are part.

Trial funders need to commission and support the coordination of research that gives trial teams evidence with which to answer their questions about *how* to achieve an ethnically diverse trial. We would prioritise work evaluating trial processes (especially recruitment and retention [39, 40]) that aim to increase involvement of specific ethnic groups and which are designed around known barriers and facilitators to the involvement of these ethnic groups in trials [18, 19, 41]. Evaluation should also include testing the impact of policies and recommendations such as ours: what difference do they really make? Once high certainty evidence is available, funders and national research infrastructure should actively facilitate its dissemination and use.

#### Policy 5: Good Clinical Practice (GCP) training should cover ethnic diversity in trials

Sponsors require almost all those involved with trials, and all those with participant-facing roles, to have mandatory research training, especially GCP training. For trials of drugs, GCP training is not just mandatory but a legal requirement in most jurisdictions.

GCP training should be modified to include material on the scientific importance of ethnic diversity in research, what can be done to support it, and what might make it less likely. It should cover the need for cultural competency training for those designing research, and those with participant-facing roles, essential diversity training that others have also highlighted [15, 16]. Some of this may be signposting, for example to sources of evidence on how to design inclusive retention strategies. We have for example started to do this in Aberdeen through discussion with colleagues at the Grampian Research Office, which offers GCP training. The reach of GCP training is so wide that it should be a cornerstone of increasing knowledge and skills around ethnically diverse trials.

## Conclusion

Agreeing targets for which ethnic groups to involve in a trial is essential but could be practically difficult. The eight STRIDE recommendations, which include minimum targets for inclusion, could help to make trials more ethnically diverse if followed. We outline five policy changes that we think would create an environment in which following the recommendations would be easier.

The recommendations, and the policies to support them, need to be implemented because more diverse trials are better, more useful science. Moreover, diversity needs to improve not only amongst those participating in trials, but those designing and running them.

Others make similar suggestions [42, 43]. To quote one of them, Khadijah Breathett, ‘These strategies may lead to a future in which healthcare is provided equitably across populations’ [42]. Quite so.

## Supplementary Information

Supplementary Material 1. Selected trials.

Supplementary Material 2. STRIDE recommendations (coding).

Supplementary Material 3. STRIDE recommendations (long).

## Data Availability

All our data and materials are available as supplements to this article, or at https://osf.io/jmqsx/ and https://www.trialforge.org/trial-diversity/include/.

## Abbreviations

COVID: Coronavirus
GCP: Good Clinical Practice
HIV: Human immunodeficiency virus
INCLUDE: Innovations in Clinical Trial Design and Delivery for the under-served
mRNA: Messenger ribonucleic acid
NICE: National Institute for Health and Care Excellence
NIHR: National Institute for Health and Care Research
NHS: National Health Service
OSF: Open Science Framework
PICO: Participants–Intervention–Comparator–Outcomes
SWAT: Study Within A Trial
UK: United Kingdom
US: United States
WHO: World Health Organization
