# Supplementary material for: How should trial teams make decisions about the proportions and diversity of the ethnic groups in their trial?
Source: Trials. 2024 Nov 15;25:768. doi: 10.1186/s13063-024-08625-5 (PMC11566274; doi:10.1186/s13063-024-08625-5)
Supplement: Supplementary file 1 — Supplementary Material 1. Selected trials. [file 13063_2024_8625_MOESM1_ESM.pdf]

Disease area	Trial title	NIHR report	Registry entry
Cancer (breast)	Exercise to prevent shoulder problems after breast cancer surgery: the PROSPER RCT	https://doi.org/10.3310/JKNZ2003	https://www.isrctn.com/ISRCTN35358984
Cancer (colorectal)	Can we save the rectum by watchful waiting or transanal surgery following (chemo)radiotherapy versus total mesorectal excision for early rectal cancer?	N/A	https://www.isrctn.com/ISRCTN14240288
Cancer (colorectal)	3-month versus 6-month adjuvant chemotherapy for patients with high-risk stage II and III colorectal cancer: 3-year follow-up of the SCOT non-inferiority RCT	https://doi.org/10.3310/hta23640	https://www.isrctn.com/ISRCTN59757862
Cancer (prostate)	Active monitoring, radical prostatectomy and radical radiotherapy in PSA-detected clinically localised prostate cancer: the ProtecT three-arm RCT	https://doi.org/10.3310/hta24370	https://www.isrctn.com/ISRCTN20141297
Cardiovascular disease	A facilitated home-based cardiac rehabilitation intervention for people with heart failure and their caregivers: a research programme including the REACH-HF RCT	https://doi.org/10.3310/pgfar09010	https://www.isrctn.com/ISRCTN86234930
Cardiovascular disease	Adrenaline to improve survival in out-of-hospital cardiac arrest: the PARAMEDIC2 RCT	https://doi.org/10.3310/hta25250	https://www.isrctn.com/ISRCTN73485024
Cardiovascular disease	An extended stroke rehabilitation service for people who have had a stroke: the EXTRAS RCT	https://doi.org/10.3310/hta24240	https://www.isrctn.com/ISRCTN45203373
Cardiovascular disease	Emergent aneurysm treatment compared with treatment on neurological improvement in patients with ruptured poor-grade aneurysmal subarachnoid haemorrhage: the TOPSAT2 RCT	https://doi.org/10.3310/eme08080	https://www.isrctn.com/ISRCTN15960635

Cardiovascular disease	OUTREACH study: Urine analysis and antihypertensive treatment	N/A	https://www.isrctn.com/ISRCTN15911100
Cardiovascular disease	A study to evaluate the benefit of medical therapy versus angiography and stenting in patients with heart attacks - The BHF SENIOR RITA Trial	N/A	https://www.isrctn.com/ISRCTN11343602
Diabetes (type 1)	A cluster randomised trial, cost-effectiveness analysis and psychosocial evaluation of insulin pump therapy compared with multiple injections during flexible intensive insulin therapy for type 1 diabetes: the REPOSE Trial	https://doi.org/10.3310/hta21200	https://www.isrctn.com/ISRCTN61215213
Diabetes (type 2)	Behavioural interventions to promote physical activity in a multiethnic population at high risk of diabetes: PROPELS three-arm RCT	https://doi.org/10.3310/hta25770	https://www.isrctn.com/ISRCTN83465245
Diabetes (type 2)	Long limb compared with standard limb Roux-en-Y gastric bypass for type 2 diabetes and obesity: the LONG LIMB RCT	https://doi.org/10.3310/eme08030	https://www.isrctn.com/ISRCTN15283219
Diabetes (type 1 and type 2)	Intravitreal aflibercept compared with panretinal photocoagulation for proliferative diabetic retinopathy: the CLARITY non-inferiority RCT	https://doi.org/10.3310/eme05050	https://www.isrctn.com/ISRCTN32207582
Maternal and infant health	When to induce labour to limit risk in pregnancy hypertension – a multicentre, randomised controlled trial	Trial is ongoing Award information including link to protocol: https://fundingawards.nihr.ac.uk/award/16/167/123	https://www.isrctn.com/ISRCTN77258279
Maternal and infant health	A randomised controlled trial evaluating the clinical and cost effectiveness of intrauterine insemination (IUI) versus in vitro fertilisation (IVF) for unexplained infertility	N/A	Trial was not funded.

Mental health	Improving mental health and reducing antipsychotic use in people with dementia in care homes: the WHELD research programme including two RCTs	https://doi.org/10.3310/pgfar08060	https://www.isrctn.com/ISRCTN62237498
Mental health	Switching antipsychotic medication to reduce sexual dysfunction in people with psychosis: the REMEDY RCT	https://doi.org/10.3310/hta24440	https://www.isrctn.com/ISRCTN12307891
Mental health	Antidepressant treatment with sertraline for adults with depressive symptoms in primary care: the PANDA research programme including RCT	https://doi.org/10.3310/pgfar07100	https://www.isrctn.com/ISRCTN84544741
Mental health	Antidepressant medication to prevent depression relapse in primary care: the ANTLER RCT	https://doi.org/10.3310/hta25690	https://www.isrctn.com/ISRCTN15969819
Mental health	Social recovery therapy for young people with emerging severe mental illness: the Prodigy RCT	https://doi.org/10.3310/hta25700	https://www.isrctn.com/ISRCTN47998710
Mental health	A health promotion intervention to improve lifestyle choices and health outcomes in people with psychosis: a research programme including the IMPaCT RCT	https://doi.org/10.3310/pgfar08010	https://www.isrctn.com/ISRCTN58667926
Smoking cessation	Nicotine replacement treatment, e-cigarettes and an online behavioural intervention to reduce relapse in recent ex-smokers: a multinational four-arm RCT	https://doi.org/10.3310/hta24680	https://www.isrctn.com/ISRCTN11111428
