# Supplementary material for: How should trial teams make decisions about the proportions and diversity of the ethnic groups in their trial?
Source: Trials. 2024 Nov 15;25:768. doi: 10.1186/s13063-024-08625-5 (PMC11566274; doi:10.1186/s13063-024-08625-5)
Supplement: Supplementary file 2 — Supplementary Material 2. STRIDE recommendations (coding). [file 13063_2024_8625_MOESM2_ESM.pdf]

Thoughts on STRIDE summaries

23/2/2023

General

The three main areas of summary are:

1. What a panel said about a particular trial re. the ethnic groups that should be involved and at what proportion.
2. What panels said in general about a) the ethnic groups that should be involved in trials that focus on particular diseases or conditions and b) the design choices for these trials irrespective of ethnicity.
3. What panels and the STRIDE team said or have learnt about the process of considering which ethnic groups a trial needs.

The summaries below focus on these three areas. Panels did sometimes discuss issues outside of these three areas and this discussion does not appear in the tables below. The text in square brackets (i.e. '[..]') allows us to link a comment in this document to one or more comments in the summary document we produced after each discussion, in other words a type of referencing system.

For example, D1: c11, c12 means Diabetes summary 1 (raw), comments 11 and 12.

Trials

Diabetes

Trial: REPOSE (type 1 diabetes) [https://www.isrctn.com/ISRCTN61215213; https://www.journalslibrary.nihr.ac.uk/hta/hta21200#/abstract]

Summary type ID	Panel suggestion	Supplementary STRIDE team suggestion
1	South Asian and Black individuals must be involved because the risk and severity of potential complications of type 1 diabetes are known to be greater for individuals from these ethnic groups.<br><br>[D1: c10]	Panel did not suggest particular percentages for this trial. From general discussion with all STRIDE panels for all trials, we suggest a default of inclusion of the specified ethnic groups at the **same proportion as is found among the population of people with the condition targeted by the trial.**<br><br>The proportion is dependent on the intended reach of the applicability of its results. A trial intending national reach should aim for national ethnic proportions by disease. A trial with more local reach could aim for proportions in its local area.
2	The trial team would need to carefully consider how they would collect ethnicity data from participants.<br><br>[D1: c8]	

2 (General)	Younger people of all ethnicities, especially those under 18, are often excluded from diabetes trials. Whether exclusion is appropriate should be carefully considered by the trial team.<br><br>The panel recognised that interventions targeting people under the age of 18 may need to be different to those targeting older people and a single trial of both would therefore be inappropriate. Nevertheless, there was a belief that exclusion was often more related to a perceived difficulty with consent or ethical procedures rather than whether the intervention could benefit younger people.<br><br>[D1: Gen c3; c6]	Trial teams may make a decision to exclude some groups for a variety of reasons (e.g. clinical care is different for, say, younger patients than for older patients, or that cultural preferences mean that this particular intervention is highly unlikely to be acceptable to some groups, for who a different intervention is needed). We recognise that such decisions will be needed in some trials and that such decisions can be justified.<br><br>The key message from the panel discussion around exclusions was to make the reason for the exclusion explicit.
2 (General)	The trial team would need to carefully consider the language support that may be needed to ensure that members of the ethnic groups important to the trial can actually participate. This requires consideration of both written translation and interpretation.<br><br>[D1: Gen c4]	

Trial: PROPELS (type 2 diabetes) [https://www.isrctn.com/ISRCTN83465245; https://www.journalslibrary.nihr.ac.uk/hta/hta25770]

Summary type ID	Panel suggestion	Supplementary STRIDE team suggestion
1	South Asian, Black African and African Caribbean individuals must be involved because the disproportionate Type 2 diabetes disease burden borne by them.<br><br>[D1: c11; c12]	Panel did not suggest particular percentages for this trial. From general discussion with all STRIDE panels for all trials, we suggest a default of inclusion of the specified ethnic groups at the **same proportion as is found among the population of people with the condition targeted by the trial.**<br><br>The proportion is dependent on the intended reach of the applicability of its results. A trial intending national reach should aim for national ethnic proportions by disease. A trial with more local reach could aim for proportions in its local
2	What is meant by South Asian needs to be defined by the trial team.<br><br>[D1: c11]	
2	The increased prevalence and disease burden of type 2 diabetes is widely recognised for South Asians but other ethnic groups with increased burden such as Black African and African Caribbean should not be forgotten.<br><br>[D1: c11; c17; c19]	
2	Interpretation of BMI by ethnicity is not straightforward. BMI thresholds vary by ethnicity and there are certainly different thresholds for South Asian participants. Using BMI as an eligibility criterion is best avoided for this reason.<br><br>[D1: c16]	
2	In trials where ethnicity is considered likely to have an effect on outcomes, stratification by ethnicity should be considered.<br><br>[D1: c19]	

2 (General)	Younger people of all ethnicities, especially those under 18, are often excluded from diabetes trials. Whether exclusion is appropriate should be carefully considered by the trial team.<br><br>The panel recognised that interventions targeting people under the age of 18 may need to be different to those targeting older people and a single trial of both would therefore be inappropriate. Nevertheless there was a belief that exclusion was often more related to a perceived difficulty with consent or ethical procedures rather than whether the intervention could benefit younger people.<br><br>[D1: Gen c3]	
2 (General)	The trial team would need to carefully consider the language support that may be needed to ensure that members of the ethnic groups important to the trial can actually participate. This requires consideration of both written translation and interpretation.<br><br>[D1: Gen c4]	

Trial: LONG LIMB (type 2 diabetes and obesity) [https://www.isrctn.com/ISRCTN15283219; https://www.journalslibrary.nihr.ac.uk/eme/eme08030#/abstract]

Summary type ID	Panel suggestion	Supplementary STRIDE team suggestion
1	Early stage trials should also consider the ethnic groups needed for the trial to be as informative as it can be.<br><br>The trial should aim to include those most at risk of type 2 diabetes and obesity. The trial population should reflect the ethnic diversity of those who are most at risk of type 2 diabetes and obesity. This would certainly include South Asian and Black individuals.<br><br>[D1: c20; c25]	Panel did not suggest particular percentages for this trial. From general discussion with all STRIDE panels for all trials, we suggest a default of inclusion of the specified ethnic groups at the **same proportion as is found among the population of people with the condition targeted by the trial.**<br><br>The proportion is dependent on the intended reach of the applicability of its results. A trial intending national reach should aim for national ethnic proportions by disease. A trial with more local reach could aim for proportions in its local
2	The extent to which being overweight is considered a problem varies by ethnic group. Trial teams working on obesity trials need to be aware of this difference, especially if groups that often do not consider being large to be a problem are those who are objectively at highest risk of poor health.<br><br>The same is true of surgery as a weight-reduction option: how acceptable it is may vary by ethnicity.<br><br>[D1: c21; c22]	

2	Obesity has a stigma among some ethnic groups and trial teams need to be aware of this when designing and running their trial.<br><br>[D1: c24]	Trials dealing with weight management need to be aware of wider social movement around weight and body size. For example a movement called 'health at every size' (HAES), is gaining support particularly with younger/more left-leaning people, including people from ethnic minority backgrounds.<br><br>Movements such as HAES mean that stigma is not only in one direction– there's also some stigma in trying to lose weight as it's seen to be conforming to patriarchal views. Young women in particular are now focusing on weight inclusivity and other measures of health (respectful care, eating for well-being, life-enhancing movement etc) and getting away from 'pathologising specific weights'. If weight loss is the goal for a trial then there will likely be some push back with groups looking into and following this and similar models.
2	The term 'person living with obesity' was considered better than calling someone obese.<br><br>[D1: c24]	Language around weight and body size and shape is complex. A different group of panel members may have preferred different terms. The key message for trial teams is that they need to be sure that the language they use in the discussions with potential and actual trial participants is alive to these sensitivities.
2 (General)	Younger people of all ethnicities, especially those under 18, are often excluded from diabetes trials. Whether exclusion is appropriate should be carefully considered by the trial team.<br><br>The panel recognised that interventions targeting people under the age of 18 may need to be different to those targeting older people and a single trial of both would therefore be inappropriate. Nevertheless there was a belief that exclusion was often more related to a perceived difficulty with consent or ethical procedures rather than whether the intervention could benefit younger people.<br><br>[D1: Gen c3]	

2 (General)	The trial team would need to carefully consider the language support that may be needed to ensure that members of the ethnic groups important to the trial can actually participate. This requires consideration of both written translation and interpretation.<br><br>[D1: Gen c4; c23]	

Trial: CLARITY (diabetic retinopathy) [https://www.isrctn.com/ISRCTN32207582; https://www.journalslibrary.nihr.ac.uk/eme/eme05050#/abstract]

Summary type ID	Panel suggestion	Supplementary STRIDE team suggestion
1		Panel did not suggest particular percentages for this trial. From general discussion with all STRIDE panels for all trials, we suggest a default of inclusion of the specified ethnic groups at the **same proportion as is found among the population of people with the condition targeted by the trial.**<br><br>The proportion is dependent on the intended reach of the applicability of its results. A trial intending national reach should aim for national ethnic proportions by disease. A trial with more local reach could aim for proportions in its local area.<br><br>[D2: c13]
2	The trial targets both type 1 and type 2 diabetes. The distribution of participants across type 1 and type diabetes would need to be monitored to avoid one or other dominating in an unexpected way. The average age was 51, which perhaps suggests more type 2 than type 1.<br><br>[D2: c2]	
2	The attention given to fertility in the eligibility criteria is likely to exclude many younger people and the implications of this for applicability need to be clear to the trial team.<br><br>[D2: c3]	
2	The vision-related criterion suggests relatively mild vision impairment but perhaps this preferentially excludes some ethnic groups more because they present later and have greater problems by the time they present.<br><br>[D2: c5]	

2	The trial team would need to carefully consider the language support that may be needed to ensure that members of the ethnic groups important to the trial can actually participate. This is particularly important because of the long list of exclusions linked to sexual issues.<br><br>Consideration of both written translation and interpretation is needed.<br><br>[D2: c4]	
2	The panel discussed payment/incentives and that these have an impact on behaviour in terms of engagement with the trial: £££ = 'I don't want to mess up their trial' and FREE = 'I want to feel better'<br><br>[D2: c12]	
2 (General)	Younger people of all ethnicities, especially those under 18, are often excluded from diabetes trials. Whether exclusion is appropriate should be carefully considered by the trial team.<br><br>The panel recognised that interventions targeting people under the age of 18 may need to be different to those targeting older people and a single trial of both would therefore be inappropriate. Nevertheless there was a belief that exclusion was often more related to a perceived difficulty with consent or ethical procedures rather than whether the intervention could benefit younger people.<br><br>[D1: Gan c3; D2: c1]	
2 (General)	Trial teams need to be aware that recruiting some groups may be easier than others and need to consider the possibility of trial spaces filling up before many from more diverse thence groups can join. Perhaps staged recruitment would be better to ensure that space remains for that diverse recruitment.<br><br>[D2: c6]	

2 (General)	The ‘ability to give informed consent’ is open to prejudice. How is the judgement made and who are recruiters going to tell about the trial?<br><br>[D2: c8]	
2 (General)	The design of trials needs to be made in light of the fact that some people will take additional treatments themselves. The trial should be transparent about other treatments, not something that can or can’t be allowed. Need to find out what participants are also using as part of the trial research.<br><br>[D2: c9]	

Cancer

Trial: SCOT trial (colorectal cancer) [https://www.isrctn.com/ISRCTN59757862; https://doi.org/10.3310/hta23640]

Summary type ID	Panel suggestion	Supplementary STRIDE team suggestion
1	The Panel recommended that the **minimum target for diverse ethnic involvement should be that the trial involves different ethnic groups at the proportions found in the most recent census data for the geographical areas where recruitment is being done**. It is important to note that demographics are changing and that e.g. census data may be old and that targets should aim to take account of current and future demographics, not past demographics.<br><br>There was also support for oversampling Black African Caribbean and South Asian individuals because of a current paucity of trial data for these groups and because data show the Black African Caribbean people are often diagnosed at a younger age and are more likely to present with Stage IV disease.<br><br>[C1: c7; c14]	
2	Over-sampling of minority groups should be the default as this is unlikely to affect the applicability of results to the majority population but will provide more data than we generally see at present for ethnic minority groups.<br><br>[C1: c11]	
2	The statistical analysis for the trial should always consider ethnicity. In other words, there should be an analysis that explores the potential for ethnicity-related effects. Without this more diverse ethnic group inclusion becomes a largely descriptive exercise.<br><br>[C1: c10]	

2	The source data used to make decisions about the ethnic groups to be involved and at what proportion should be clear in trial reports. The level of uncertainty of these data should also be made clear.<br><br>This is important to avoid poor quality data effectively hard-wiring discrimination into future trials.<br><br>[C1: c3]	
2	The generally poor quality of ethnicity data with regard to prevalence and disease severity was noted.<br><br>[C1: c4]	
2	Criteria linked to the ethnic groups to be involved should be part of the formal trial eligibility criteria. This would raise the importance and the attention given to non-clinical eligibility criteria.<br><br>[C1: c6]	
2	Trial teams should use the most detailed census data that they can get old of and these can be very local and detailed. This will also help to plan where to place trial recruitment sites.<br><br>[C1: c9]	

Trial: STAR-TREK (rectal cancer) [https://www.isrctn.com/ISRCTN14240288; https://www.cancerresearchuk.org/about-cancer/find-a-clinical-trial/a-trial-looking-at-surgery-or-different-types-of-radiotherapy-for-rectal-cancer-star-trec#undefined]

Summary type ID	Panel suggestion	Supplementary STRIDE team suggestion
1	The Panel recommended that the **minimum target for diverse ethnic involvement should be that the trial involves different ethnic groups at the proportions found in the most recent census data for the geographical areas where recruitment is being done**. The data available on prevalence and severity were not good enough to suggest anything else.<br><br>There was also support for oversampling of ethnic minority groups but which groups to target in particular was unclear because of poor prevalence and severity data.<br><br>[C1: c19]	
2	This trial starts with people having already made a decision that they would like to keep some of their rectum.<br><br>The trial results will be relevant for those individuals who have decided to consider options to retain part of their rectum and they may be ethnically different to all people with rectal cancer but we have no data to say whether they do or not. The trial team should monitor who says yes and no to taking part to see if the two groups are ethnically different.<br><br>[C1: c15; c16]	Collecting ethnicity information for people who say no to taking part in a trial is likely to present both practical and ethical approval challenges. The key point is that it is important to consider how closely the diversity of the trial population matches the population the trial team decided it needed prior to starting recruitment and retention. Doing this may not need to know the ethnicity of those who said no: a comparison of those who said yes against the target population may be enough.<br><br>This point made by the panel also highlights that the difference between intervention and comparator may be far less clear to potential participants than it is to the trial team. People considering major surgery for cancer may see little difference in alternative surgical approaches: from the potential participant's perspective it's all surgery.<br><br>Trial teams could routinely embed checking of intervention/ comparator understanding, something that is likely to be a [illegible]

2	The choice mentioned above (i.e. to retain part of the rectum) could also differ by other characteristics such as age and gender. These too should be monitored.<br><br>[C1: c17]	

Trial: PROSPER (breast cancer) [https://www.isrctn.com/ISRCTN35358984; https://doi.org/10.3310/JKNZ2003]

Summary type ID	Panel suggestion	Supplementary STRIDE team suggestion
1		Panel did not suggest particular percentages for this trial. From general discussion with all STRIDE panels for all trials, we suggest a default of inclusion of the specified ethnic groups at the **same proportion as is found among the population of people with the condition targeted by the trial.**<br><br>The proportion is dependent on the intended reach of the applicability of its results. A trial intending national reach should aim for national ethnic proportions by disease. A trial with more local reach could aim for proportions in its local area.<br><br>[C2: c14]
2	The statistical analysis for the trial should always consider ethnicity. In other words, there should be an analysis that explores the potential for ethnicity-related effects. Without this more diverse ethnic group inclusion becomes a largely descriptive exercise.<br><br>[C2: c5]	
2	The source data used to make decisions about the ethnic groups to be involved and at what proportion should be clear in trial reports. The level of uncertainty of these data should also be made clear.<br><br>This is important to avoid poor quality data effectively hard-wiring discrimination into future trials.<br><br>[C2: c7]	

2	The range of experiences individuals from different ethnic groups have may be best obtained by good patient and public involvement during trial planning and design.<br><br>[C2: c8]	
2	The panel raised the possibility of over-sampling but noted the challenge of deciding what the levels should be. Regardless of what they are, those levels should have some sort of error bar or range around them. There is plenty of uncertainty when choosing these levels.<br><br>[C2: c9]	
2 (General)	If there are differences between ethnic groups, these differences are more likely to be due to ethnic minority experiences of poor care and low expectation than of biological differences between people of different ethnic groups. Different groups may not accept the treatment to the same extent and that sort of difference is what we should be paying most attention to because it will be far greater (in most cases) than any biological difference.<br><br>[C2: c10; c11]	
2 (General)	The data that are really needed (updated of breast cancer surgery by ethnicity) are lacking, which makes it hard to plan a trial of this sort. The data we have is prevalence of breast cancer, which is not the starting point for this trial.<br><br>Other characteristics than ethnicity (e.g. income, gender) may also be important.<br><br>[C2: c2; c4]	

Title: PROTECT (prostate cancer) [https://www.isrctn.com/ISRCTN20141297; https://doi.org/10.3310/hta24370]

Summary type ID	Panel suggestion	Supplementary STRIDE team suggestion
1		Panel did not suggest particular percentages for this trial. From general discussion with all STRIDE panels for all trials, we suggest a default of inclusion of the specified ethnic groups at the **same proportion as is found among the population of people with the condition targeted by the trial.**<br><br>The proportion is dependent on the intended reach of the applicability of its results. A trial intending national reach should aim for national ethnic proportions by disease. A trial with more local reach could aim for proportions in its local area.<br><br>The lack of data to inform discussions about prostate cancer and ethnicity is a substantial problem.<br><br>[C2: c20]
2	Trial teams should compare the ethnic diversity of their trial population (i.e. those who take up the offer to take part in a trial) with those with the condition in the community more generally. They may be different.<br><br>[C2: 16]	
2	The design of the trial needs to be discussed prior to the trial start with individuals who understand the perspectives, values and preferences of ethnic groups that need to be part of the trial. These discussions may need to be in languages other than English.<br><br>[C2: c17]	

2	Discussions with individuals from different ethnic groups may need to include family members (partners particularly) who are often crucial in persuading men to seek medical help. Panel members mentioned the phrase ‘toxic masculinity’ as a barrier to seeking help, and family/partners can help to overcome this.<br><br>[C2: c18]	

Cardiovascular disease

Trial: REACH-HF (cardiac rehab after heart failure) [https://www.isrctn.com/ISRCTN86234930; https://doi.org/10.3310/pgfar09010]

Summary type ID	Panel suggestion	Supplementary STRIDE team suggestion
1	The Panel recommended that the **minimum target for diverse ethnic involvement should be that the trial involves different ethnic groups at the proportions found in the most recent census data for the geographical areas where recruitment is being done**.<br><br>The overall ethnic diversity of the trial should be census levels.<br><br>[CVD1: c8]	
2	The attractiveness of lifestyle and physical activity change is not highly regarding by all ethnic groups (the panel mentioned South Asian and Arab culture as giving less regard to these). Attitudes may be more negative in older generations but their views greatly influence younger people too. Increasing physical activity is not universally seen as a good thing even by younger people.<br><br>[CVD1: 2]	
2	For trials involving South Asian individuals, changes to diet need to be alive to the views of other family members (and especially the matriarch of the family) on low calorie food. Low calorie food (e.g. a milkshake) may not fit the cultural expectations of what the cook of the family wanted to provide and that can lead to retention problems.<br><br>[CVD1: c3]	
2	Trial teams need to explicitly think about ethnicity when selecting their trial recruitment sites. Trial teams need to think about who the trial needs and where do they live.<br><br>[C2: c5]	

2	Home-based, self-management interventions are unlikely to be equally attractive to all ethnic groups. Trials teams need to monitor who says yes and who stays in a trial by ethnicity.<br><br>This was a theme that came up a number of times: careful monitoring of who enters a trial and who stays with it by ethnicity.<br><br>[CVD1: c6]	The acceptability of self-management is likely to be disease-specific. In cardiovascular disease, which generally carries little stigma, home-based self-management may be acceptable to many people. On the other hand, for mental health where stigma is high across many ethnic groups, home-based self-management ties into the social stigma and the 'outing' of people as living with mental ill health. Such interventions may then be much less acceptable to potential participants.
2	Trial teams need to think carefully about whether the ethnic groups a trial needs may lead to a conclusion that separate trials evaluating different interventions are needed.<br><br>Some interventions may be predictably unacceptable to some ethnic groups and then the best thing is to acknowledge this and accept that different interventions (and trials) are needed. Some of the information about whether separate trials will be needed could come from feasibility studies perhaps.<br><br>[CVD1: c11]	

Trial: PARAMEDIC (out of hospital heart attack) [https://www.isrctn.com/ISRCTN73485024; https://doi.org/10.3310/hta25250]

Summary type ID	Panel suggestion	Supplementary STRIDE team suggestion
1		Panel did not suggest particular percentages for this trial. From general discussion with all STRIDE panels for all trials, we suggest a default of inclusion of the specified ethnic groups at the **same proportion as is found among the population of people with the condition targeted by the trial.**<br><br>The proportion is dependent on the intended reach of the applicability of its results. A trial intending national reach should aim for national ethnic proportions by disease. A trial with more local reach could aim for proportions in its local area.
2	The Panel recommended that careful monitoring of who enters a trial and who stays with it by ethnicity is important. It would be useful to compare the ethnicity of the trial population with the ethnicity of the local area from which a trial site is recruiting.<br><br>The trial relies on someone calling for an ambulance and some ethnic groups (South Asians were mentioned) may be less likely to call for an ambulance, or recognise the signs of a heart attack too late for the trial to be relevant.<br><br>CVD1: c11; c12]	
2	Although there was no consent in the emergency setting itself, consent was sought from survivors or their families later on. This too could lead to differences by ethnicity so also points to monitoring who is in the trial compared to the ethnicity in the local area.<br><br>How information is provided for this consent process should also consider cultural differences linked to ethnicity.<br><br>[CVD1: c13]	

2	Trial teams need to explicitly think about what other treatments a person may be taking post heart attack. Some ethnic groups may supplement health service treatment with other forms of treatment, which may or may not be useful (or harmful). This points to an assessment of outcome by ethnicity and monitoring what additional treatments participants are taking.<br><br>[CVD1: c15]	

Trial: BHF SENIO RITA (heart attack) [https://www.isrctn.com/ISRCTN11343602]

Summary type ID	Panel suggestion	Supplementary STRIDE team suggestion
1	The Panel recommended that the **minimum target for diverse ethnic involvement should be that the trial involves different ethnic groups at the proportions found in the most recent census data for the geographical areas where recruitment is being done**.<br><br>The overall ethnic diversity of the trial should be census levels.<br><br>[CVD1: c22]	
2	The Panel thought that age was probably being used as a proxy for frailty comorbidities and that this would likely lead to less representation of ethnic minority groups because similar frailty/comorbidty would occur earlier in some ethnic minority groups (e.g. South Asians). Trial teams would need to consider this during their design and conduct.<br><br>CVD1: c17]	
2	If an age cut-off is used as in this trial, it is important to consider where the ethnicity of those in the data that support the choice of the cut-off point. The panel suspected that the data supporting the cut-off of 75 would come from a predominantly white population, which means its relevance to other ethnic groups is uncertain.<br><br>[CVD1: c18]	

2	The decision to approach a person for the trial relies on clinician judgement and as with other trials, where recruitment decisions are down to human judgement there is scope for bias.<br><br>The panel thought this would be a problem for older Asian women in particular where risk, though real, was likely to be considered higher than it actually is.<br><br>[CVD1: c19]	

Trial: EXTRAS (stroke) [https://www.isrctn.com/ISRCTN45203373; https://doi.org/10.3310/hta24240]

Summary type ID	Panel suggestion	Supplementary STRIDE team suggestion
1	The Panel recommended that the **minimum target for diverse ethnic involvement should be that the trial involves different ethnic groups at the proportions found in the most recent census data for the geographical areas where recruitment is being done**.<br><br>There was support for over-sampling some ethnic groups, particularly Black individuals (women especially) and South Asians.<br><br>Decisions around the ethnic groups to include need epidemiological data and that these data should be as local to a recruitment site as they can be. Context is important.<br><br>[CVD2: c13; c3; c8]	All panels emphasised the importance of paying attention to ethnic diversity in the area where recruitment is being done.<br><br>As a general strategy, we would suggest that trial teams consider the ethnic groups needed by their trial and then explicitly choose trial sites that are located in areas that would support involvement of those ethnic groups.<br><br>In other words, ethnicity would be a direct factor in choosing the location of trial sites, not only something to monitor after sites are selected.
2	The data available to inform decisions is often not sufficiently detailed enough to give this sort of information. Issues around the data collection and coding of the data are important if research and health systems are to be improved with regard to the care given to ethnic minority individuals.<br><br>[CVD2: c5]	
2	The Panel wondered whether the younger end of the age range (i.e. 18 - 40) of all ethnicities would be less likely to engage with rehabilitation.<br><br>CVD2: c6]	

2	The Panel thought that the weight of the trial would be with older people because stroke affects older people in the main. The trial team therefore needs to know who gets stroke by ethnicity and which of them would be candidates for rehabilitation of the sort described in this trial. Given the point mentioned earlier about younger groups, for this intervention perhaps it should be targeting older people mostly.<br><br>[CVD2: c9]	
2	Over-sampling of Black individuals and South Asian individuals may be useful, in large part in direct recognition of the paucity of data linked to individuals from these ethnic groups in research generally.<br><br>The Panel recognised the need to talk to statisticians about this with regard to the implications for analysis and sample size.<br><br>[CVD2: c10]	
2	Household structure may have a role with regard to the support people from some ethnic groups may have. Multigenerational households may provide more social support, something more likely to happen in South Asian cultures. This may mean that some ethnic groups are more likely to have a carer.<br><br>The influence of generations within a house will affect the decisions of other generations within the same house (four example the older generation may influence the younger). Those same younger individuals may take a different decision if they were living in their own house, away from the older generation (and vice versa).<br><br>[CVD2: c11]	

Trial: TOPSAT2 (haemorrhage) [https://www.isrctn.com/ISRCTN15960635; https://doi.org/10.3310/eme08080]

Summary type ID	Panel suggestion	Supplementary STRIDE team suggestion
1	The Panel recommended that the **minimum target for diverse ethnic involvement should be that the trial involves different ethnic groups at the proportions found in the most recent census data for the geographical areas where recruitment is being done**.<br><br>Decisions around the ethnic groups to include need epidemiological data and that these data should be as local to a recruitment site as they can be. Context is important.<br><br>The Panel introduced the idea of including stratification by ethnicity into the randomisation system to ensure that equal numbers of particular ethnic groups were allocated to intervention and comparison groups. This is especially important because the number of individuals from some groups is likely to be small.<br><br>[CVD2: c3; c15; c17]	
2	The data available to inform our decisions is often not sufficiently detailed enough to give this sort of information. Issues around the data collection and coding of the data are important if research and health systems are to be improved with regard to the care given to ethnic minority individuals.<br><br>Data for the type of stroke is important because, for example, haemorrhagic stroke is more common in Black people and Chinese people. This sort of information should directly inform who is in the trial.<br><br>[CVD2: c5; c14]	

Trial: OUTREACH (hypertension) [https://www.isrctn.com/ISRCTN15911100]

Summary type ID	Panel suggestion	Supplementary STRIDE team suggestion
1	The Panel struggled to make clear recommendations about which ethnic groups should be involved but tended to the direction of involvement at same proportion as is found among the population of people with the condition targeted by the trial.<br><br>The proportion is dependent on the intended reach of the applicability of its results. A trial intending national reach should aim for national ethnic proportions by disease. A trial with more local reach could aim for proportions in its local area.<br><br>[CVD2: c25]	
2	Black individuals were highlighted as being least likely to have treatment and don't engaged the same extent with medication. Members of the panel had seen Black individuals who have hypertension that is extremely worrying but they themselves are unconcerned, they don't have symptoms as such and therefore do not see the need to treatment.<br><br>Some of this could be linked to religious beliefs. Ignoring faith when trying to involve Black individuals would be a mistake, not only for this type of trial.<br><br>[CVD2: c19]	
2	It would be worth considering stratification by faith/belief as well as ethnicity.<br><br>[CVD2: c20]	

2	Household structure may have a role with regard to the support people from some ethnic groups may have. Multigenerational households may provide more social support, something more likely to happen in South Asian cultures. This may mean that some ethnic groups are more likely to have a carer.<br><br>The influence of generations within a house will affect the decisions of other generations within the same house (four example the older generation may influence the younger). Those same younger individuals may take a different decision if they were living in their own house, away from the older generation (and vice versa).<br><br>[CVD2: c22]	
2	Trial teams need to explicitly think about what other treatments a person may be taking. Some ethnic groups may supplement health service treatment with other forms of treatment, which may or may not be useful (or harmful). This points to an assessment of outcome by ethnicity and monitoring what additional treatments participants are taking.<br><br>[CVD2: c24]	
2	The Panel wondered whether part of the trial design process should ask an open question of individuals along the lines of what would you do if your hypertension was not as it should be? What strategies would help you? How would these fit with your health belief systems?<br><br>The answers may help to tailor both interventions design and delivery, and information provision.<br><br>[CVD2: c23]	

Mental health

Trial: WHELD (psychosis and dementia) [https://www.isrctn.com/ISRCTN62237498; https://doi.org/10.3310/pgfar08060]

Summary type ID	Panel suggestion	Supplementary STRIDE team suggestion
1	The trial should set a minimum level for ethnic groups and then look to see where these people are living across the country (i.e. it should drive the selection of recruitment sites).<br><br>[MH1: c9]	Panel did not suggest particular percentages for this trial. From general discussion with all STRIDE panels for all trials, we suggest a default of inclusion of the specified ethnic groups at the **same proportion as is found among the population of people with the condition targeted by the trial.**<br><br>The proportion is dependent on the intended reach of the applicability of its results. A trial intending national reach should aim for national ethnic proportions by disease. A trial with more local reach could aim for proportions in its local area.
2	The trial is based in care homes and the Panel was unsure as to how well the care home setting looked like the population at large with regard to ethnicity (opinion was divided). Regardless, the starting point for any trial team would be to consider whether the setting for their trial is the right setting with regard to the ethnicity of those who have the clinical problem targeted by the trial.<br><br>The Panel discussed a layering of questions that a trial team would need to ask. This would start with something like who are the people with dementia? Followed by and of these which people are in care homes? And of these what proportion are overmedicated? All questions would need to include consideration of ethnicity.<br><br>[MH1: c3; c6; c10]	

2	It is likely that the ethnic diversity of care homes will vary across the country and this should directly inform decisions about where to place recruitment sites.<br><br>[MH1: c7]	
2	The trial is about over-treatment but for ethnic minority individuals the Panel wondered whether the problem was more likely to be under-treatment.<br><br>[MH1: c4]	

Trial: REMEDY (sexual dysfunction in people with psychosis) [https://www.isrctn.com/ISRCTN15969819; https://www.journalslibrary.nihr.ac.uk/hta/hta24440/#/abstract]

Summary type ID	Panel suggestion	Supplementary STRIDE team suggestion
1	The trial should set a minimum level for ethnic groups based on who takes these drugs and the likelihood of sexual dysfunction and then look to see where these people are living across the country (i.e. it should drive the selection of recruitment sites).<br><br>[MH1: c19]	Panel did not suggest particular percentages for this trial. From general discussion with all STRIDE panels for all trials, we suggest a default of inclusion of the specified ethnic groups at the **same proportion as is found among the population of people with the condition targeted by the trial.**<br><br>The proportion is dependent on the intended reach of the applicability of its results. A trial intending national reach should aim for national ethnic proportions by disease. A trial with more local reach could aim for proportions in its local area.
2	The trial would need to ask the a set of layered questions about who are the people who have sexual dysfunction after taking these drugs and what is their ethnicity? Gender is likely to be important too.<br><br>[MH1: c16; c17]	
2	Trial teams need to be cautious about assuming that sensitivity around the trial topic maps onto ethnicity in the way they think unless they have spoken to people from those ethnic groups as part of trial design and their assumptions confirmed.<br><br>[MH1: c15]	
2	Trials teams may need to consider whether separate trials that involve different interventions tailored to particular ethnic groups might be necessary. This may be a general consideration where the trial is tackling something very sensitive and cultural values may play a strong role. These challenges need to be tackled though, otherwise little will change.<br><br>[MH1: c20; c22]	

2	Language is a particularly important consideration for a trial dealing with a sensitive topic, it needs to be exactly right. Funders need to be willing to pay for language support, including interpretation where needed.<br><br>[MH1: c21; c24]	
2 (general)	The Panel talked around how the proportion of individuals from particular ethnic groups might be increased and noted work from health economists that can be summarised as which participant will add most value to the study?<br><br>If the study has already a large number of individuals from one ethnic group then adding one more is of less value to the study's ability to generate information than to add an individual from a different ethnic group. This might be a consideration that all trials, not just this one, could consider.<br><br>[MH1: c23]	

Trial: IMPACT (psychosis) [https://www.isrctn.com/ISRCTN58667926; https://doi.org/10.3310/pgfar08010]

Summary type ID	Panel suggestion	Supplementary STRIDE team suggestion
1	The key problem with arriving at particular ethnic groups and proportions is the lack of data on condition and ethnicity to inform this decision.<br><br>[MH1: c30]	Panel did not suggest particular percentages for this trial. From general discussion with all STRIDE panels for all trials, we suggest a default of inclusion of the specified ethnic groups at the **same proportion as is found among the population of people with the condition targeted by the trial.**<br><br>The proportion is dependent on the intended reach of the applicability of its results. A trial intending national reach should aim for national ethnic proportions by disease. A trial with more local reach could aim for proportions in its local area.
2	The trial would need to ask the a set of layered questions about who are the people who has psychosis and what is their ethnicity and who are registered with the Care Approach Programme (an eligibility criterion)? Is this intervention design (and this eligibility criterion) suitable for all ethnic groups?<br><br>[MH1: c28; c29]	

Trial: PANDA (depression) [https://www.isrctn.com/ISRCTN84544741; https://doi.org/10.3310/pgfar07100]

Summary type ID	Panel suggestion	Supplementary STRIDE team suggestion
1	Broad inclusion of ethnic groups across the trial population (because all ethnic groups are susceptible to depression) was raised during the discussion.<br><br>[MH2: c9; c17]	Panel did not suggest particular percentages for this trial. From general discussion with all STRIDE panels for all trials, we suggest a default of inclusion of the specified ethnic groups at the **same proportion as is found among the population of people with the condition targeted by the trial.**<br><br>The proportion is dependent on the intended reach of the applicability of its results. A trial intending national reach should aim for national ethnic proportions by disease. A trial with more local reach could aim for proportions in its local area.
2	Trial teams need to consider that the starting point of patients may differ by ethnicity.<br><br>For example in some South Asian groups individuals are often using alternative remedies, herbal remedies for example. If these do not help then they will eventually seek medical help. If health professionals treat the person as though this is their first attempted treatment it is likely that the person will go away feeling they have been ignored. Their situation is already more advanced because they have already exhausted a range of alternative remedies. This is not their first presentation.<br><br>[MH2: c4]	
2	Trials teams need to be aware that mental health diagnoses are themselves stigmatised and contested, which compounds problems for individuals who also come from ethnic minority backgrounds.<br><br>[MH2: c7]	

2	Sertraline can be harder to come off and has some stigma around it particularly among younger people as a drug that has withdrawal problems if switching from it to something else. It is unclear whether this is also a perception held across ethnic groups.<br><br>[MH2: c16]	
2 (general)	Trial teams needs to consider how people view research. People from minority groups are more likely to think of research as them being experimented on and without their consent based in historical examples of exactly that and people have long memories.<br><br>Non-health issues (e.g. the UK's Windrush scandal) also influences health-care decision-making now. Why would minority groups want to help a system that is always using them?<br><br>[MH2: c11]	
2 (general)	Research is also seen as being very elitist also an issue the people of colour. Researchers themselves are often not very representative of society as a whole.<br><br>[MH2: c12]	

2 (general)	Researchers need to be open to all people and trials should be targeting x% from this group and y% from that group and stopped if they don't meet those targets. There needs to be a consequence for not working to improve the diversity of trials.<br><br>[MH2: c13]	While having targets linked to ethnicity in trials is likely to increase ethnic diversity in trials (which is needed) these targets may themselves come across to some individuals in ethnic minority groups as ethnic minority people being used again to further someone else's career, usually white researchers.<br><br>This perspective is linked to direct experience by many ethnic minority individuals of insufficient effort being put into then ensuring that everyone that needs the treatment or an intervention can engage with it and receive that treatment or intervention.<br><br>Building trust and effective, honest communication about what greater ethnic diversity in a trial may mean for those communities once the trial is complete will be important. Simply saying to potential participants that the trial has targets may do as much harm to trust as it does good.
2 (general)	The conversation with an individual to discuss possible involvement in the trial needs to be open and explain exactly what will happen and what the potential benefits are. What will you get? What is the offer?<br><br>[MH2: c14]	

Trial: ANTLER (depression relapse) [https://www.isrctn.com/ISRCTN15969819; https://doi.org/10.3310/hta25690]

Summary type ID	Panel suggestion	Supplementary STRIDE team suggestion
1	Broad inclusion of ethnic groups across the trial population because all ethnic groups are susceptible to depression<br><br>[MH2: c19; c24]	Panel did not suggest particular percentages for this trial. From general discussion with all STRIDE panels for all trials, we suggest a default of inclusion of the specified ethnic groups at the **same proportion as is found among the population of people with the condition targeted by the trial.**<br><br>The proportion is dependent on the intended reach of the applicability of its results. A trial intending national reach should aim for national ethnic proportions by disease. A trial with more local reach could aim for proportions in its local area.
2	The starting point of the trial is a diagnosis of depression and be receiving treatment for it. This itself may vary by ethnicity, being harder for some groups to get.<br><br>[MH2: c18]	
2	Recruitment of some ethnic groups (e.g. Black men) may be easier using non-health service channels. Barbershops were mentioned as one route for recruiting Black men.<br><br>[MH2: c20]	
2	The language used (not just world language but the style and complexity of language) needs careful consideration. Literacy is likely be more of a problem for some (South Asians were mentioned). Organised religion may have a role in recruiting from some ethnic groups and provides a non-health service route.<br><br>[MH2: c3; c21]	

Trial: Prodigy (young people with emerging severe mental illness) [https://www.isrctn.com/ISRCTN47998710; https://doi.org/10.3310/hta25700]

Summary type ID	Panel suggestion	Supplementary STRIDE team suggestion
1	Broad inclusion of ethnic groups across the trial population because all ethnic groups are susceptible to mental illness.<br><br>The poor availability of data to inform decisions about who should be in the trial was noted. There was doubt as to whether a single intervention that was acceptable to all ethnic groups was possible.<br><br>[MH2: c35]	Panel did not suggest particular percentages for this trial. From general discussion with all STRIDE panels for all trials, we suggest a default of inclusion of the specified ethnic groups at the **same proportion as is found among the population of people with the condition targeted by the trial.**<br><br>The proportion is dependent on the intended reach of the applicability of its results. A trial intending national reach should aim for national ethnic proportions by disease. A trial with more local reach could aim for proportions in its local area.
2	The framing of this trial suggested a particular view of what was a useful way of spending time and that this is likely a majority white view and potentially middle-class too.<br><br>For example the outcome of the trial is linked to what the team calls structured activity, which includes economic activity (i.e. working), school and social activities such as going to the cinema. It is far from clear that all ethnic groups share the same views of what those structured activities should be especially the non-work activities.<br><br>[MH2: c25]	

2	Socio-economic status is important for this trial and is intertwined with ethnicity in that some ethnic groups are more likely to be socio-economically disadvantaged than others.<br><br>Irrespective of what people might want to do (i.e. if helped by the intervention) their ability to do the sorts of activities, particularly social ones suggested by the trial team, may be limited by lack of money. This makes it less likely that some ethnic groups (and more socio-economically disadvantaged people within all ethnic groups) will be less able to take part.<br><br>It would have been better for the program itself to provide the activities free of charge. Depending on where activities take place, there is a risk that a person's mental illness may be made more visible in the local community. The stigma of mental illness (common across all ethnic groups) and the risk of being identified as a person with mental illness may be enough to prevent participation.<br><br>Intersectionality is important.<br><br>[MH2: c27; c28; c31]	
2	The main trial outcome was considered a potential problem. There may be a clinical recovery and social recovery that form part of the outcome, and there is judgement required as to what constitutes recovery. The outcome chosen is a value laden judgement.<br><br>[MH2: c30]	

Maternal health

Trial: WILL (early-term delivery) [https://www.isrctn.com/ISRCTN77258279; https://fundingawards.nihr.ac.uk/award/16/167/123]

Summary type ID	Panel suggestion	Supplementary STRIDE team suggestion
1	The panel noted the increased levels of hypertension (and uncontrolled hypertension) in non-Hispanic Black women, suggesting these women as an important group for the trial. It was also noted that there are other groups with increased hypertension perhaps not linked to ethnicity, but e.g. socio-economic status. The ethnic background of many of these individuals may well be white. Age was also mentioned. In other words intersectionality was mentioned.<br><br>In summary, the trial should target ethnicities at high risk, in particular Black women.<br><br>[Mat: c2; c4; c10]	
2	The panel discussed how people from some ethnic groups access care later, which leads to a greater chance of problems. This was particularly true for ethnic minority groups. How to interact with women in a culturally competent way, and overcoming system and structural problems (and language potentially) are challenges that trial teams need to consider explicitly during design and conduct.<br><br>[Mat: c5]	
2 (general)	Engagement with the health care system, including maternity care, depends on how the service engages with you. In other words 'How seen' and how validated (i.e. that your perspective is listened to and considered important) you feel after interactions. If the feeling you are left with is that you are neither seen nor validated, you are less likely to continue engaging.<br><br>[Mat: c7]	

Trial: UNI (IVF treatment) [unfunded trial so no registration link]

Summary type ID	Panel suggestion	Supplementary STRIDE team suggestion
1	The Panel struggled to reach a firm conclusion around the ethnic groups involved and at what proportion but tended towards the trial involving different ethnic groups at the proportions found in the most recent census data for the geographical areas where recruitment is being done.<br><br>Providing a clear recommendation was difficult because of the lack of (easily accessible) data on the background rate of infertility and endometriosis by ethnicity. This makes it difficult to say who should be in the trial.<br><br>The panel also noted the difficulty regarding using diagnostic information. Historically white women have been more diagnosed as having endometriosis than other ethnic groups. This means the background rate data are likely to under-represent endometriosis among other ethnic groups. There was general agreement that this is a clear problem for trialists attempting to design a trial that represents the ethnic groups most affected.<br><br>The Panel thought over-sampling of some ethnic groups would be likely to be helpful.<br><br>[Mat: c11; c15]	
2	The panel discussed how people from some ethnic groups access care later, which leads to a greater chance of problems. This was particularly true for ethnic minority groups. How to interact with women in a culturally competent way, and overcoming system and structural problems (and language potentially) are challenges that trial teams need to consider explicitly during design and conduct.<br><br>[Mat: c5]	

2	The panel though over-sampling would be sensible to increase the amount of data available on historically under-served ethnic groups and which would increase the certainty of what could be said from the data collected for those groups.<br><br>Moreover, this should be linked to eligibility criteria and perhaps an extra table in trial reports highlighting numbers of individuals from particular ethnic groups linked to eligibility (and need).<br><br>[Mat: c12]	
2 (general)	Engagement with the health care system, including maternity care, depends on how the service engages with you. In other words ‘How seen’ and how validated (i.e. that your perspective is listened to and considered important) you feel after interactions. If the feeling you are left with is that you are neither seen nor validated, you are less likely to continue engaging.<br><br>[Mat: c7]	

Smoking cessation

Trial: Relapse prevention trial [https://www.isrctn.com/ISRCTNISRCTN11111428; https://www.journalslibrary.nihr.ac.uk/hta/hta24680/#/abstract]

Summary type ID	Panel suggestion	Supplementary STRIDE team suggestion
1	The Panel struggled to come down to particular percentages for particular ethnic groups. The closest we came for older Pakistani men who were considered an important group as were individuals (particularly men) from countries such as Poland, Albania is and Romania where smoking is much more prevalent and therefore more likely here in the UK when people move. The panel did not suggest percentages.<br><br>The thrust of the conversation would support aiming to involve people at the level of smoking in the community, which is why for example Polish men would be an important group for the trial in excess of their representation in the community as a whole.<br><br>[Sm: c14]	
2	The trial used the UK Stop Smoking Service, which is likely to lead to a different ethnic diversity than the population of smokers who would like to stop smoking. Trial teams should consider the implications of the setting and eligibility criteria their trials have to ensure that they understand the potential impact on ethnic diversity within the trial.<br><br>Public contributor comments during the panel discussion suggested that the UK Stop Smoking Service was not widely known among at least some ethnic groups and would anyway be unlikely to be used by those groups.<br><br>[Sm: c2]	

2	Over-sampling was discussed as a way of reducing uncertainty regarding subgroup analysis by ethnicity.<br><br>[Sm: c10]	
2	Intersectionality was mentioned. It was difficult for the panel to separate out other characteristics from ethnicity, for example age and gender. Other factors, for example socio-economic status, were also mentioned, particularly because smoking is a costly habit. This suggested that this stage of identifying who needs to be in the trial may also need to consider at the very least age and gender in addition ethnicity.<br><br>Public contributors highlighted differences between how interventions affect different groups, in this case age groups. Older people stop smoking only when they go to the doctor for emerging health issues. Government interventions like age limits and bans etc impacted young people, but rarely the older groups - the older people have houses and cars to smoke in; they're at a less social point in their lives but they are also willing to sacrifice social experiences because they are already addicted.<br><br>[Sm: c6; c12]	
2	People who smoke are not all the same. A parallel was drawn to use of alcohol where people could effectively be more problem smokers and would need different support services those who were not such heavy smokers. Whether a single intervention is suitable for all irrespective ethnicity was unclear.<br><br>[Sm: c7]	
2 (General)	It is important to define what is meant by ethnic minority or diverse ethnic groups because not all people will see themselves as part of a minority group even if others do. The example given was of someone from Eastern Europe may not consider themselves to be in an ethnic minority group but for some trials (such as this one on smoking) they may be key group to involve.<br><br>[Sm: c11]	

2 (General)	The ability to take part in smoking cessation trials should also be linked explicitly to the cost of smoking. Could be useful for other interventions/ disease areas and situations that are costly - e.g. having a physical disability.<br><br>[Sm: c13]	

Process insights

Summary type ID	Panel suggestion	Supplementary STRIDE team suggestion
3		Panels need to comprise more than the trial team. Each panel needs people with lived experience of the disease/condition, clinical experience of treating the disease or condition, as well as trial team members with experience of designing and running trials.<br><br>An essential starting point for all panel discussions is to have no consideration of the practicalities of involving people: the question is who needs to be in the trial. How those people can be involved is a question for later. Trial teams tend to think about the practicalities immediately, which is one reason why there needs to be other people in the discussion.<br><br>STRIDE team c1
3		Having ethnic diversity among patient and public contributors with lived experience is essential to a successful panel. These individuals speak with authority on both the condition but also how people from their ethnic group view the condition, treatment options and the proposed trial. These perspectives are crucial if the trial is to be inclusive.<br><br>It is unlikely to be feasible for a trial team to involve patient and public contributors from all the ethnic groups important for their trial in panel discussions. However, ensuring that the most important perspectives are directly represented will improve the trial teams ability to design a trial that can involve individuals from the ethnic groups needed for the trial to be as useful and relevant as it can be.<br><br>STRIDE team c2

3		Discussions are more productive when the trial team has found information regarding disease/condition prevalence and severity by ethnicity.<br><br>Sadly, this information cannot always be found even after an extensive search. But the aim should be to have as much data about prevalence, severity, and progression by ethnicity as possible before holding the meeting to discuss which groups must be in the trial and at what proportion.<br><br>STRIDE team c3
3		Discussions need time.<br><br>We would suggest a minimum of 2 hours, which allows time to introduce the trial, the disease and its implications and the information around disease prevalence and severity by ethnicity.<br><br>STRIDE team c4
3		The discussions are most productive when focused on which groups are needed rather than how difficult it might be to involve those groups. A discussion about practicalities and strategies will be needed but it isn't the place to start.<br><br>[Sm: c4]

3		Selecting the ethnic groups needed for a trial and at what proportion is a hard task. To make the task easier, we suggest that:<br><br>**The default starting point should be that ethnic groups are included at the same proportion as is found among the population of people with the condition targeted by the trial.**<br><br>This gives a concrete starting point, which can then be modified if discussion suggests it is necessary.<br><br>**If there are insufficient data on disease prevalence, severity and progression by ethnicity, the next default starting point should be that ethnic groups are involved at the proportions found in the most recent census data for the geographical areas where then trial is being done.**<br><br>The proportion is dependent on the intended reach of the applicability of its results. A trial intending national reach for its results should aim for national ethnic proportions by disease. A trial with more local reach could aim for proportions in its local area, which may be very different to the national proportions by ethnicity.<br><br>We suggest that ethnicity should be a factor in deciding where to place trial sites. In other words, once the ethnic groups needed in a trial has been decided, the next step would be to look for where people in these ethnic groups live and to place trial sites as close to them as possible.<br><br>STRIDE team c5
3	There should be a discussion of the need to over-sample some populations.<br><br>[C1: c10]	
3	Alternative phrases for over-sampling include ‘representative sampling’ and ‘reflective sampling’.<br><br>[C1: c12]	

3	Research teams and panels should themselves aim to be more diverse, not only in ethnicity but other characteristics. Seeing researchers from your own community reassures members of the public in many cases.<br><br>[C2: c12; c13]	
3	Education was considered key to improving awareness of these issues amongst majority populations and researchers. It was noted that the problem concerned not only ethnicity but other issues such as gender and socio-economic status. Explicit discussions of the sort panels have is part of this education.<br><br>[C2: c13]	
