# Supplementary material for: How should trial teams make decisions about the proportions and diversity of the ethnic groups in their trial?
Source: Trials. 2024 Nov 15;25:768. doi: 10.1186/s13063-024-08625-5 (PMC11566274; doi:10.1186/s13063-024-08625-5)
Supplement: Supplementary file 3 — Supplementary Material 3. STRIDE recommendations (long). [file 13063_2024_8625_MOESM3_ESM.pdf]

How should a trial team make a decision about the ethnic groups that are important for their trial?

Background

The STRIDE project (https://www.abdn.ac.uk/hsru/what-we-do/research/projects/stride-supporting-recruitment-and-retention-improvements-for-diverse-ethnicities-283) involved discussion in late 2022/early 2023 with ten panels (see last page). These panels discussed a total of 23 trials with the aim of saying which ethnic groups the trials needed to include and what proportion of the trial population they should be.

While considering a particular trial, panels sometimes provided advice on how a trial team might have answered this question. Additionally, having listened to ideas and suggestions from many individuals and panels across multiple trials and disease areas, the STRIDE team itself reflected on what might be useful advice for trial teams grappling with how to decide which ethnic groups their trial needs.

This document packages that advice into eight recommendations.

The Recommendations

1. Trial teams need to be more diverse *[C2: c12; c13]*

Trial teams should themselves aim to be more diverse, not only in terms of ethnicity but other characteristics too. Seeing researchers from your own community reassures many members of the public that research is relevant to them.

Education was considered key to improving awareness of these issues amongst majority populations and researchers. It was noted that the problem concerned not only ethnicity but other issues such as gender and socio-economic status. The discussions that trial teams have with patient and public contributors from diverse backgrounds was considered part of this education.

How should a trial team make a decision about the ethnic groups that are important for their trial?

2. Trial teams need the perspectives of people from diverse ethnic communities with lived experience of the condition or disease1 *[STRIDE team [c1]]*

Having ethnic diversity among patient and public contributors with lived experience is essential. These individuals speak with authority on both the condition but also how people from their ethnic group view the condition, treatment options and the proposed trial. These perspectives are crucial if the trial is to be inclusive.

Trial teams need to consider cultural differences and how comfortable patient and public contributors may be in a group setting. For example, not everyone is comfortable with revealing their name or showing their face on camera during video meetings. It may be that trial teams need to have one-to-one conversations with people to ensure perspectives and experiences are shared fully.

It is unlikely to be feasible for a trial team to involve patient and public contributors from all the ethnic groups important for their trial. However, ensuring that key perspectives are directly represented will improve the trial team's ability to design a trial that is as useful and relevant as possible to all those who could potentially benefit.

Public and patient contributors should always be paid for their contributions.

3. Discussions need time *[STRIDE team [c4]]*

We would suggest a minimum of two hours, which allows time to introduce the trial (we found using PICO (Participants–Intervention–Comparator–Outcomes) worked well for this), the disease or condition and its implications and the data around disease prevalence, severity and progression by ethnicity.

Discussions will need patient and public contributors (Recommendation 2) and these individuals should be reimbursed for their time. This means that trial teams will need to budget for their participation. Patient and public contributors sometimes prefer to receive material on paper through the post rather than electronically, which will also have a cost. Finally, some or all of the discussion may sometimes be better done face-to-face but outside the researchers' own environment (e.g. at a local community centre), which may also require funding.

4. Discussions are more productive with data on disease or condition prevalence, severity and progression by ethnicity *[STRIDE team [c3]]*

Discussions about which ethnic groups to involve in a trial are more straightforward when informed by data on disease or condition prevalence, severity and progression by ethnicity.

Sadly these data cannot always be found even after an extensive search. Alternatively the data may exist but are not publicly available. However, the aim should be to have as much data about disease or condition prevalence, severity and progression by ethnicity as possible before discussing which ethnic groups should be involved in the trial.

It should be noted though that where data are available, these data are likely to have been gathered from health systems where inequalities exist and recording of ethnicity information may be poor. The data may therefore give a skewed or incomplete picture: it is important to combine data with the first-hand perspectives of people from diverse ethnic communities (Recommendation 2).

Having a target range for the participation of a particular ethnic group (i.e. between A% and B%) rather than a single percentage (i.e. A%) will help to account for uncertainty, as will considering over-sampling (Recommendation 7).

5. Don't think about how to recruit and retain people *[STRIDE team [c2]; Sm: c4]*

Thinking about the practicalities of *how* to recruit and retain people is a question for later: the question for now is which ethnic groups need to be in the trial. Researchers in trial teams tend to think about the practicalities of recruitment and retention immediately but that isn't what this stage of trial design is about. This is one reason why there needs to be more than researchers in the discussion.

How should a trial team make a decision about the ethnic groups that are important for their trial?

6. If in doubt, use the STRIDE defaults for ethnic group inclusion *[STRIDE team [c5]]*

Selecting the ethnic groups needed for a trial and at what proportion is a hard task. Where a team cannot reach a conclusion with regard to the ethnic groups needed, or perhaps lacks the time and/or resources to have a full discussion, the STRIDE panel discussions suggest adopting the following default inclusion position:

a. The minimum target is that ethnic groups are included at the same proportion as is found among the population of people with the condition targeted by the trial. The proportion is dependent on the intended reach of the applicability of trial results. A trial intending national reach should aim for national ethnic proportions by disease. A trial with more local reach could aim for proportions in the relevant geographical areas.

Where disease data by ethnicity do not exist, or cannot be obtained, the STRIDE panel discussions suggest adopting the following default inclusion position:

b. The minimum target is that ethnic groups are included at the same proportion as is found in the most recent census data. The proportion is dependent on the intended reach of the applicability of trial results. A trial intending national reach should use national census data. A trial with more local reach could aim for census proportions in the relevant geographical areas.

7. Consider over-sampling some ethnic groups[2] *[C1: c10; C1: c12]*

Involving some ethnic minority groups at proportions seen in the general population or at proportions seen for a particular disease or condition may mean that the number of individuals involved is still low. The ability to draw conclusions from these numbers may be limited.

The trial team could consider over-sampling these ethnic groups so that they are over-represented within the trial relative to their representation within the disease or condition, or the general population. This will increase the number of individuals from these ethnic groups in the trial and may improve the team’s ability to draw conclusions from their data.

Doing this is unlikely to reduce the certainly around conclusions drawn for majority ethnic populations unless the over-sampling is very substantial (in which case the team may wish to consider whether the trial should be targeted *only* at that over-sampled group).

8. Think about ethnicity when choosing trial sites *[STRIDE team [c5]]*

The ethnic groups a trial needs should influence decisions about where to place trial sites. Once the ethnic groups needed in a trial has been decided, the next step should be to look for where people in those ethnic groups live and to place trial sites as close to them as possible. The use of geographically localised data on ethnicity (e.g. census data) will help trial teams to make these decisions.

Notes

1. These recommendations imply that by involving a more ethnically diverse trial population, the trial will be able to say useful things about how different ethnic groups engaged with the trial and, potentially, whether outcomes varied for different ethnic groups. Trying, but failing, to involve a particular ethnic group clearly tells you something about the acceptability and utility of a trial intervention and its delivery for that ethnic group. This sort of descriptive analysis is unlikely to be controversial. Sub-group analysis focused on effect estimate differences by ethnicity is more likely to make researchers feel uneasy. Even with over-sampling (Recommendation 7), sub-groups are unlikely to be formally powered and making them so would in almost all cases lead to a prohibitively large trial. Our view is that sub-group analysis should always be pre-planned, done together with the trial statistician and should focus on looking for signals of difference rather than proof of benefit or harm for one group or another. The uncertainly around conclusions should always be acknowledged.
2. 'Over-sampling' was not the only term suggested during our discussions: other terms were 'representative sampling' and 'reflective sampling'. We have chosen 'over-sampling' instead of these alternatives because we think it better conveys what the process aims to do: produce trial populations where some groups, by design, are represented to a greater extent in the trial population than they are in populations outside the trial.

TRIAL FORGE

How should a trial team make a decision about the ethnic groups that are important for their trial?

Key messages

- Decisions around the ethnic groups needed for a particular trial need to be made by the trial team together with people from diverse ethnic communities with lived experience of the disease or condition and professionals with experience of treating the disease or condition.
- Where the trial team cannot reach a conclusion with regard to the ethnic groups needed, our discussions in STRIDE suggest adopting the following default inclusion position:
    a. The minimum target is that **ethnic groups are included at the same proportion as is found among the population of people with the condition targeted by the trial.** The proportion is dependent on the intended reach of the applicability of trial results. A trial intending national reach should aim for national ethnic proportions by disease. A trial with more local reach could aim for proportions in the relevant geographical areas.
- Where **disease data by ethnicity do not exist, or cannot be obtained,** our discussions in STRIDE suggest adopting the following default inclusion position:
    b. The minimum target is that **ethnic groups are included at the same proportion as is found in the most recent census data**. The proportion is dependent on the intended reach of the applicability of trial results. A trial intending national reach should use national census data. A trial with more local reach could aim for census proportions in the relevant geographical areas.
- Ethnicity should be a factor in deciding where to place trial sites. Once the ethnic groups needed in a trial has been decided, the next step should be to look for where people in these ethnic groups live and to place trial sites as close to them as possible. The use of geographically localised data on ethnicity (e.g. census data) will help trial teams to make these decisions

The panel members

Each of the ten panels had expertise relevant to the clinical area being discussed. These clinical areas were:

1. Bowel, breast and prostate cancer
2. Diabetes (types 1 and 2)
3. Cardiovascular disease
4. Mental health
5. Maternal and infant health
6. Smoking cessation

The 40 panel members were drawn from across the UK and Ireland and were a mix of people with clinical expertise, trial and methodological expertise, lived experience of the clinical condition, experience of representing diverse ethnic communities in discussions about health, experience of funding trials in the clinical area and health policy experience. Generally, at least two members of the project team were present. In all panels, the project team members aimed to facilitate or summarise discussion, not lead it.
